# Therapeutic benefits of recombinant alpha1-antitrypsin IgG1 Fc-fusion protein in experimental emphysema

**DOI:** 10.1186/s12931-021-01784-y

**Published:** 2021-07-16

**Authors:** Katsuyuki Takeda, Soo-Hyun Kim, Anthony Joetham, Irina Petrache, Erwin W. Gelfand

**Affiliations:** 1 Division of Cell Biology, Department of Pediatrics, National Jewish Health, Denver, CO USA; 2 College of Veterinary Medicine, Konkuk University, Seoul, South Korea; 3 Division of Pulmonary, Critical Care & Sleep Medicine, Department of Medicine, National Jewish Health, Denver, CO USA; 4 Kyoritsu-Onsen Hospital, 1-39-1 Hirano, Kawanishi, 666-0121 Japan

**Keywords:** α-1 antitrypsin, Emphysema, Mouse, Elastase, Cigarette smoke, Dendritic cells

## Abstract

**Background:**

Alpha-1 antitrypsin (AAT) is a major serine protease inhibitor. AAT deficiency (AATD) is a genetic disorder characterized by early-onset severe emphysema. In well-selected AATD patients, therapy with plasma-derived AAT (pAAT), “augmentation therapy”, provides modest clinical improvement but is perceived as cumbersome with weekly intravenous infusions. Using mouse models of emphysema, we compared the effects of a recombinant AAT-IgG1 Fc-fusion protein (AAT-Fc), which is expected to have a longer half-life following infusion, to those of pAAT.

**Methods:**

In an elastase model of emphysema, mice received a single intratracheal instillation of porcine pancreatic elastase (PPE) or human leucocyte elastase (hLE). AAT-Fc, pAAT, or vehicle was administered intraperitoneally 1 day prior to or 3 weeks following elastase instillation. Lung function and histology assessments were performed at 7 and 32 days after elastase instillation. In a cigarette smoke (CS) model of emphysema, mice were exposed to CS daily, 5 days a week, for 6 months and AAT-Fc, pAAT, or vehicle were administered every 10 days during the last 3 months of CS exposure. Assessments were performed 3 days after the last CS exposure. Immune responses to lung elastin peptide (EP) and the effects of AAT-Fc or pAAT treatment on dendritic cell (DC) function were determined ex vivo.

**Results:**

Both elastase instillation and CS exposure triggered emphysema-like alveolar enlargement, increased lung compliance, and increased markers of inflammation compared to controls. Administration of AAT-Fc either prior to or following elastase instillation or during CS exposure provided greater protection than pAAT against alveolar enlargement, lung dysfunction, and airway inflammation. When challenged ex vivo with EP, spleen mononuclear cells from elastase-exposed mice exhibited dose-dependent production of IFNγ and IL-17, suggesting immune reactivity. In co-culture experiments with splenic CD4^+^ T cells isolated from elastase-exposed mice, AAT-Fc treatment prior to EP-priming of bone marrow-derived dendritic cells inhibited the production of IFNγ and IL-17.

**Conclusions:**

Compared to pAAT, AAT-Fc more effectively prevented or attenuated elastase- and CS-induced models of emphysema. These effects were associated with immunomodulatory effects on DC activity. AAT-Fc may provide a therapeutic option to individuals with AATD- and CS-induced emphysema.

**Supplementary Information:**

The online version contains supplementary material available at 10.1186/s12931-021-01784-y.

## Background

Alpha-1 antitrypsin (AAT) is a major serine protease plasma inhibitor of neutrophil elastase [1]. In the United States, an estimated at 5.64 million individuals are AAT-deficient (AATD) [2]. The main clinical feature of AATD is early-onset emphysema. Exogenous repletion of AAT, “augmentation therapy”, is recommended for select patients with emphysema, with weekly intravenous infusions of AAT purified from pooled human plasma (pAAT) [3, 4]. However, only modest clinical benefits of augmentation therapy have been reported, and these infusions have been associated with side effects related to infusion of high amounts of protein [3, 4]. In addition, augmentation therapy is recognized as inconvenient because of the need for frequent infusions and high costs [3, 5]. AATD patients with severe and progressive emphysema may require lung transplantation, which carries major risks of morbidity and mortality [5]. Therefore, new therapies for AATD remain a critical unmet need.

In addition to inhibiting elastase, AAT has been shown to act as a potent immunomodulator [1, 6–9]. AAT treatment may be beneficial in various autoimmune diseases such as type-1 diabetes, rheumatoid arthritis, systemic lupus erythematosus, or graft versus host disease in pre-clinical animal models and clinical studies [10, 11]. One mechanism by which AAT may exert immunomodulatory effects is through suppression of dendritic cell (DC) function [10].

Compared to pAAT, which is derived from pooled human plasma, AAT-IgG1 Fc-fusion protein (AAT-Fc) is a synthesized recombinant protein. As a synthesized and fusion protein, it would be expected to reduce costs and decrease the risk of adverse events compared to pAAT [12]. The fusion of AAT with Fc is expected to extend the half-life of the protein when compared to pAAT, with comparable levels of elastase inhibition, and prolonging the biologic activity of exogenously administered AAT [13]. Indeed, a similar AAT fused with Fc has been shown to have a longer half-life in serum as well as in the airways compared to pAAT [14]. The Fc portion fused to AAT is also expected to bind to Fc receptors, which are widely expressed on inflammatory cells, including DCs, modulating their activity [15].

To compare the effects of AAT-Fc in preclinical models, two mouse models of emphysema-like disease were used. Because an imbalance in activity between the proteinase and the anti-proteinase (AAT) drives emphysema pathogenesis in AATD [3], an elastase-induced model was applied. In addition, because cigarette smoking is a major risk factor for emphysema in AATD [2], a chronic cigarette smoking (CS) exposure model was also employed. In the elastase-induced models, we used porcine pancreatic elastase (PPE) as well as human leucocyte elastase (hLE). Following a single elastase instillation in mice, emphysematous changes such as alveolar enlargement develop and progress gradually for several months [16, 17]. To demonstrate the benefits of AAT-Fc, in addition to lung function parameters, elastin autoimmunity and DC function were monitored. Autoimmunity towards lung elastin fragments [18–20] and activation of DCs [21–23] have been shown to contribute to the pathogenesis of emphysema. Using these approaches, we identified the superiority of AAT-Fc compared to pAAT in protecting lung tissue in these experimental models of emphysema.

## Methods

### Animals

Mice (C57Bl/6) were purchased from Jackson Laboratories (Bar Harbor, ME). All mice were housed under specific pathogen-free conditions. Age-matched, equal numbers of male or female mice were used in all experiments. When analyzed by gender, in a multi-way ANOVA, no sex differences were noticed in the results. Experiments were conducted under a protocol approved by the Institutional Animal Care and Use Committee of National Jewish Health.

### Development and prevention of elastase-induced emphysema

To develop elastase-induced emphysema, 6–8-week old male or female mice were anesthetized with isoflurane and PPE was instilled via the trachea. PPE (120 U/kg) (Elastin Products Company, Owensville, MO) was dissolved in 40 μL of physiological saline solution (saline) prior to instillation. PPE was instilled once, as it was previously demonstrated by us and by other laboratories that a single PPE intratracheal instillation with this dose led to significant emphysematous changes in the lungs of mice [15, 24]. In some experiments, hLE (700 U/kg) (Elastin Products Company) was used. Control mice received saline alone. Seven, 21, or 32 days after elastase administration, lung function was measured. Briefly, mice received an overdose of pentobarbital by intraperitoneal (i.p.) injection and the inserted tracheal cannula was attached to a FlexiVent system (Scireq, Montreal, Canada) to ventilate and analyze lung function. Pressure–volume curves were generated and static compliance (Cst) and total lung capacity (TLC) were calculated using FlexiVent software.

After lung function measurements, lung tissues were collected following inflation at constant pressure (15 cmH_2_O for 10 min) with 10% buffered formalin for further morphometric analyses to determine the degree of emphysematous changes. Slides of left lung tissue were stained with hematoxylin–eosin and analyzed for mean linear intercept (MLI) according to the guidelines of the American Thoracic Society/European Respiratory Society [25]. The slide images of an entire lung section excluding airways and vessels were captured using a microscope (BX40; Olympus America, Melville, NY) equipped with a digital camera (Q-color 3; Olympus America, Center Valley, PA). Mean-free distance was quantified as MLI in the captured images by randomly set test lines using NIH Image J software (available at http://imagej.nih.gov/ij/download.html). Values of alveolar surface area (S) were also calculated with MLI and TLC as described previously [26]. Bronchoalveolar lavage (BAL) was performed with 1 mL of Hanks’ balanced salt solution in a subset of mice after airway function measurements but prior to lung tissue sampling, and cells in BAL fluid were counted and differentiated as described previously [27].

### CS-induced emphysema

Eight-week old male or female mice were exposed to whole smoke generated from research grade 3R4F cigarettes (Univ. of Kentucky) using the TE-10 smoking chamber (Teague Enterprises, Davis, CA). The machine was adjusted to generate a mixture of side-stream smoke (89%) and mainstream smoke (11%). Chamber atmosphere was monitored for total suspended particulates with concentrations of 90–110 mg/m^3^, respectively. Control mice were kept in a filtered air environment. Mice received CS continuously for 5 h/day, 5 days/week for 6 months. Three days after the last CS exposure, analyses of lung function and lung morphometry were performed as described for the elastase-induced emphysema model.

### Reagents preparation

AAT-Fc was purchased from YBDY Biotechnology (Seoul, Korea). AAT-Fc was generated by expressing recombinant AAT and fusing the intact AAT gene to the constant region of IgG1 to generate soluble recombinant AAT-Fc protein. The recombinant AAT-Fc protein was produced in Chinese hamster ovary cells and purified using mini-protein A affinity *chromatography* [28]. According to the manufacturer’s catalogue, the purity of AAT-Fc was 90%. Two mg/kg (body weight) of AAT-Fc was dissolved in 200 µl of PBS and administered by i.p. injection. Other groups of mice received 2 mg/kg of pAAT (Prolastin C; Grifols USA, Los Angeles, CA), 2 mg/kg of recombinant human IgG Fc (EMD Millipore, Billerica, MA), or PBS as a vehicle by i.p. injection. Mice received treatments 1 day prior to or 21 days after elastase instillation. In CS-exposure experiments, mice received 2 mg/kg of AAT-Fc, pAAT, IgG Fc, or vehicle alone by i.p. injection every 10 days during the last 3 months of CS exposure. As the anti-proteinase activity of AAT was expected to prevent elastase-induced lung tissue damage when administered prior to elastase instillation, IgG Fc was not used as a control in this set of experiments.

### Preparation of spleen cells

To determine immune responses against lung elastin, spleen mononuclear cells (MNCs) from PPE-treated mice on day 21 were isolated by Histopaque 1083 (Sigma-Aldrich, St. Louis, MO) as described previously [26]. Cells were washed, counted, and suspended in complete RPMI 1640 tissue culture medium (Mediatech Celgro, Manassas, VA), containing heat-inactivated fetal calf serum (FCS 10%; Sigma), L-glutamine (5 mM), α-mercaptoethanol (2 mM), Hepes buffer (15 mM), penicillin (100 units/ml), and streptomycin (100 µg/ml) (all from Invitrogen). Cells (8 × 10^6^/mL) were cultured with 2, 10, or 50 µg/mL mouse lung elastin peptide (EP, Elastin Products Company) for 4 days and IFNγ and IL-17 levels in culture supernatants were measured by ELISA (eBioscience, San Diego, CA).

### AAT-Fc treatment effects on dendritic cells

DCs were derived from bone marrow of naive mice following culture with 10 ng/mL of mouse GM-CSF and IL-4 (both from Peprotech, Rocky Hill, NJ) for 1 week as described previously [29]. Cultured cells were identified as DCs; cell surface expression of CD11c and MHC Class II was greater than 98%. DCs (4 × 10^4^ cells) were suspended in RPMI culture medium and treated with 1, 10, or 100 µm of AAT-Fc or pAAT. Twenty-four hours later, DCs were washed and cultured with mouse lung elastin peptide (50 µg/mL). Following treatment with PPE, on day 21 CD4^+^ or CD8^+^ T cells were purified from spleen cells with magnetic beads (EasySep, STEMCELL Technologies, Vancouver, Canada) and 4 × 10^5^ purified CD4^+^ or CD8^+^ T cells were co-cultured with prepared DCs.

### Statistical analysis

Values for all measurements were expressed as means ± SEM. For comparisons between multiple groups, the Tukey–Kramer test was used. Nonparametric analyses, using the Mann–Whitney U test or Kruskal–Wallis test, were also applied to confirm that statistical differences remained significant, even if the underlying distribution was uncertain. The *p*-value for significance was set at less than 0.05.

## Results

### Effect of AAT-Fc administration prior to elastase instillation

**Fig. 1:**
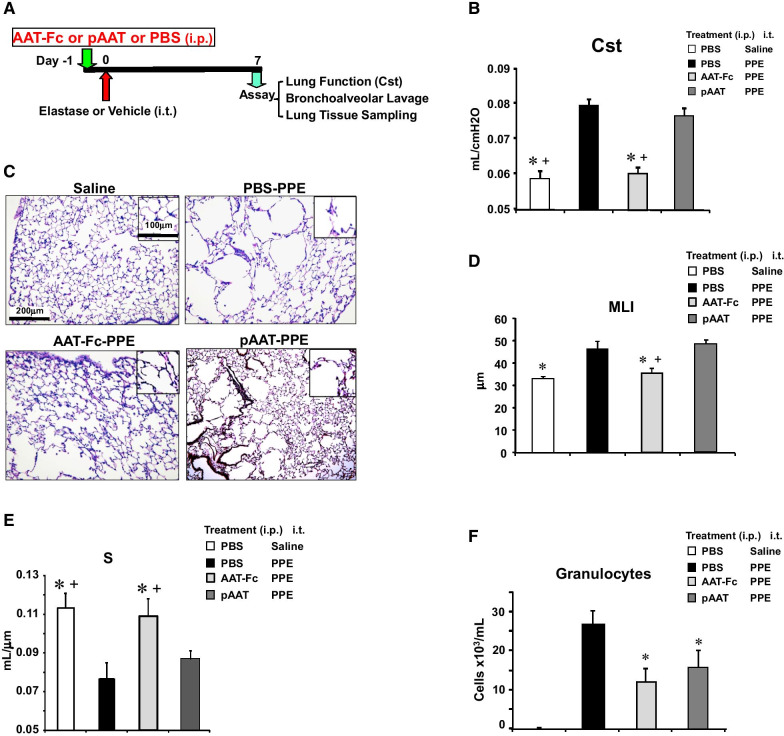
Experimental protocol to investigate the prophylactic effects of AAT-Fc on elastase-induced airspace enlargement and lung inflammation (**A**). AAT-Fc or control treatments were administered 1 day prior to PPE instillation and the effects in the lungs were analyzed 1 week later. Cst values (**B**), representative photomicrographs of lung tissue sections (**C**), MLI (**D**), S (**E**) and numbers of granulocytes in BAL fluid (**F**) were compared in mice that received saline instillation (saline), vehicle (PBS-PPE), AAT-Fc (AAT-Fc-PPE) or pAAT (pAAT-PPE) treatment prior to PPE. n = 8 in each group. *p < 0.05 vs. PBS-PPE by Tukey–Kramer test, + p < 0.05 vs. pAAT-PPE by Tukey–Kramer test

To investigate preventative effects, AAT-Fc or pAAT was administered prior to elastase instillation followed 1 week later by lung analyses (Fig. 1A). PPE instillation significantly increased granulocytic inflammation in BAL fluid and resulted in airspace enlargement with disruption of alveolar walls, as determined by increased Cst and MLI and decreased S values (Fig. 1B–E). AAT-Fc or pAAT treatment significantly reduced numbers of granulocytes by 56% and 40% respectively in the BAL fluid of PPE-instilled mice (Fig. 1F). Unlike pAAT administration, AAT-Fc treatment prior to PPE instillation significantly inhibited alveolar enlargement (24% decrease in MLI and 30% increase in S values) and lung dysfunction (23% decrease in Cst) induced by elastase (Fig. 1B–E). hLE instillation induced more modest airway inflammation (data not shown) but caused alveolar enlargement with increased MLI and decreased S values and increased Cst. Pretreatment with AAT-Fc, but not pAAT prevented the development of airspace enlargement following hLE instillation (Additional file 1: Figure S1).

### Effect of AAT-Fc aministration following elastase instillation

**Fig. 2:**
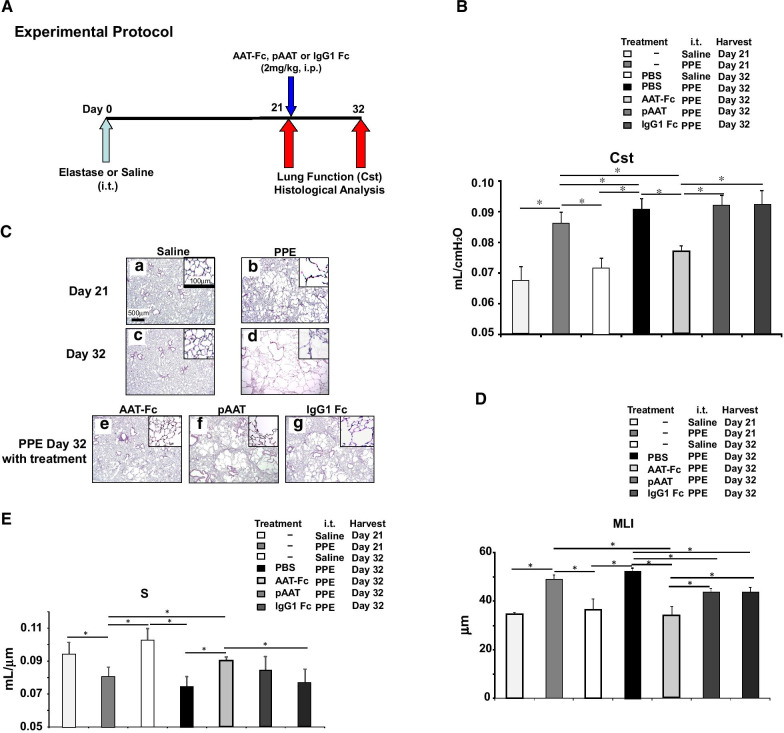
Experimental protocol to assess the effects of AAT-Fc following elastase instillation (**A**). Mice received AAT-Fc, pAAT, IgG1-Fc or vehicle 21 days after PPE instillation and the effects in the lungs were analyzed 32 days after PPE (AAT Fc PPE day 32, pAAT PPE day 32, IgG1-Fc PPE day 32, and PBS PPE day 32, respectively). A control group received saline instillation and lungs were analyzed 32 days later. Separate groups of mice were analyzed 21 days after saline or PPE instillation without any treatment (saline day 21 and PPE day 21, respectively). Cst values (**B**), lung tissue sections (**C**), MLI (**D**), and alveolar surface area (S) (**E**) values were compared. n = 8 in each group. *p < 0.05 by Tukey–Kramer test

To investigate the effect of treatment when administered after the onset of lung injury, AAT-Fc, pAAT, or controls were administered 21 days after a single PPE instillation (Fig. 2A). At 21 days after PPE instillation and then 11 days after treatment (day 32 following PPE), lung function and histopathological analyses were performed. As shown in Fig. 2B–E, at day 21 after a single PPE instillation, lung pathology and alveolar enlargement were clearly evident. When analyses were carried out on day 32, the administration of AAT-Fc (on day 21) significantly decreased Cst and MLI values and increased S values (15% and 35% decrease and 18% increase, respectively) compared with vehicle treatment, and these effects were significantly more pronounced than following pAAT or IgG1-Fc treatment. Moreover, only AAT-Fc treatment improved lung dysfunction (10% improvement in Cst and 30% improvement in MLI and increased values of 8%) compared to day 21 values following PPE instillation. These data suggested that AAT-Fc treatment not only attenuated the progression of lung damage, but reversed the pathologic events initiated by PPE.

### Effect of AAT-Fc treatment on CS-induced emphysema

**Fig. 3:**
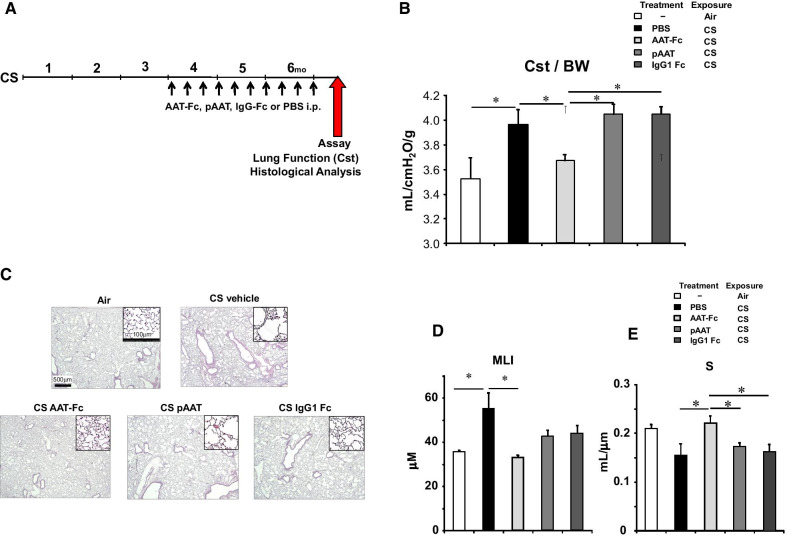
Experimental protocol to assess the effects of AAT-Fc following cigarette smoke exposure (**A**). During the last 3 months of 6-month long CS exposure, mice received AAT-Fc, pAAT, IgG1-Fc, or PBS as a vehicle control every 10 days (Cs AAT-Fc, CS pAAT, CS IgG1-Fc, or CS vehicle, respectively). A control group of mice were exposed to ambient air (Air). Cst values (**B**), lung tissue sections (**C**), MLI (**D**), and alveolar surface area (S) (**E**) values were compared between each group of mice. n = 8 in each group. *p < 0.05 by Tukey–Kramer test

Mice received AAT-Fc, pAAT, IgG1-Fc, or PBS as a vehicle control every 10 days during the last 3 months of the 6-month CS exposure (Fig. 3A). Lung function testing in CS-exposed mice revealed significant reductions in Cst following treatment with AAT-Fc (14% increase) but not following pAAT or IgG1-Fc treatment which were similar to vehicle treatment (Fig. 3B). Histopathological analyses demonstrated significant improvements in destruction of alveolar septa, MLI and S values following AAT-Fc treatment (40% decrease and 48% increase, respectively), but not following pAAT or IgG1-Fc treatments (Fig. 3C–E).

### Effect of AAT-Fc treatment on elastin autoimmunity and DC function

**Fig. 4:**
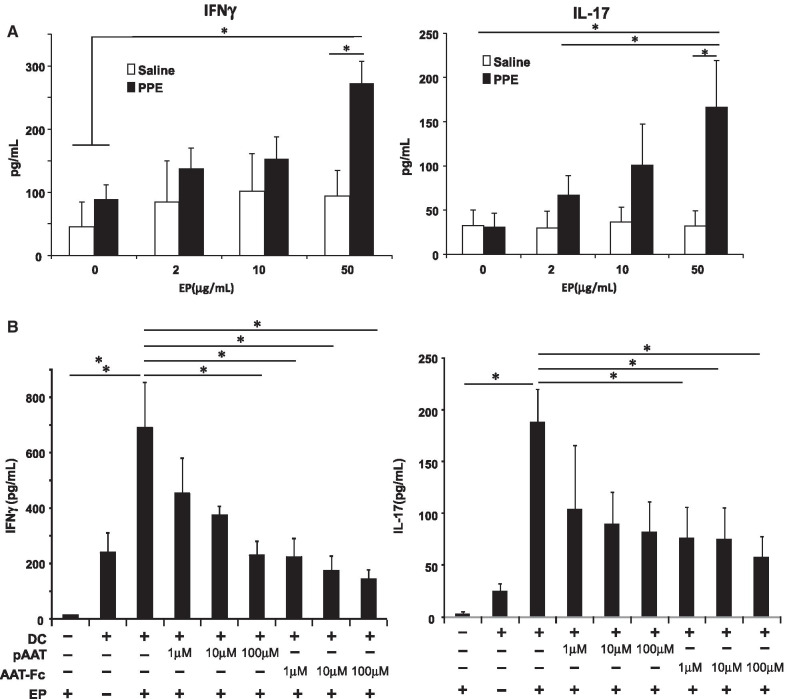
Effects of AAT Fc treatment on elastin immunity and DC function. Spleen MNCs from mice that received elastase were co-cultured with EP and culture supernatants were analyzed for IFNγ or IL-17 production (**A**). The effects of AAT-Fc or pAAT pre-treatment of bone marrow derived, EP-primed DCs on CD4^+^ T cell production of IFNγ or IL-17 (**B**). n = 6 in each group. *p < 0.05 by Tukey–Kramer test

Despite transient enzymatic activity of PPE following intratracheal instillation [30], alveolar enlargement progressed for several months [16, 17]. The sustained lung destruction may be explained by autoimmunity towards lung tissue components such as EP which is induced by elastase. In a first set experiments, immune responses towards EP in spleen MNCs were determined in vitro. As shown in Fig. 4A, IFNγ and IL-17 levels were increased following incubation of spleen MNCs with EP. These increases in cytokine levels were only seen following incubation of spleen MNCs from PPE-treated mice (data not shown), implying that a subset of spleen MNCs from PPE-treated mice developed an immune response to EP.

In light of the suggested immune response to EP and the importance of DC antigen-presentation, in a second set of experiments, bone marrow-derived DCs were treated with AAT-Fc or pAAT prior to EP exposure and co-cultured with spleen CD4^+^ T cells isolated from mice that received PPE. As shown in Fig. 4B, spleen CD4^+^ T cells co-cultured with EP-primed DCs resulted in increased levels of IFNγ or IL-17 production. These levels were decreased following co-culture of these CD4^+^ T cells with EP-primed DCs which were pretreated with AAT-Fc or pAAT compared to co-culturing with EP-primed DCs pre-treated with vehicle. The inhibitory effects of AAT-Fc treatment on DCs were greater than those on pAAT-treated DCs. Co-culturing of CD8^+^ T cells from PPE-treated mice with DCs showed no increases in IFNγ or IL-17 production (data not shown).

## Discussion

In the present study, the therapeutic benefits of recombinant AAT-Fc treatment in two experimental models of emphysema were investigated and compared to effects seen following plasma-derived, pAAT treatment. Beneficial effects were demonstrated at two stages of elastase-induced lung damage, either when administered prior to elastase instillation, suggesting prophylactic activity, and when administered after elastase, with attenuation of progression of lung injury accompanied by reversal of existing damage. AAT-Fc treatment prior to PPE instillation inhibited the development of airway inflammation and increases in airspace enlargement, both physiologically and morphologically. AAT-Fc also showed protective effects on lung tissue damage following human elastase instillation. When treatments were initiated three weeks after PPE instillation, AAT-Fc not only reduced parameters correlating with airspace enlargement, but returned these parameters to their near-normal state. In all aspects, the effects of AAT-Fc were greater than those seen with pAAT or control, IgG1-Fc. The benefits of AAT-Fc treatment were also shown in CS-exposed mice with improvements in airspace enlargement. Spleen MNCs isolated from PPE-treated mice and exposed to EP produced IFNγ and IL-17. In these studies, the inhibitory effects of AAT-Fc on cytokine production were shown to reside on EP primed-DCs when co-cultured with CD4^+^ T cells.

Intravenous augmentation therapy with pAAT has, to date, been the only specific treatment for AATD patients with advanced emphysema [3, 4]. However, studies revealed only modest effects of this therapy; this treatment slowed progression of emphysema as measured by lung density with computed tomography or total lung capacity, but not when measured by functional residual capacity [31]. Following a single administration of PPE or hLE, we demonstrated the prophylactic effects of AAT-Fc on airway inflammation and alveolar enlargement, whereas pAAT failed to prevent alveolar enlargement. As damage of lung tissue at an early time-point following elastase instillation is thought to be induced by proteinase activity, these results support potent anti-protease activities of AAT-Fc in the prevention of elastase-induced lung damage.

Similar robust beneficial effects of AAT, especially with AAT-Fc, were seen in our model of CS-exposure. AAT treatment has been shown to be beneficial in other mouse models of emphysema, including those induced by vascular endothelial growth factor receptor blockade [32] or by CS exposure. Relatively high doses of pAAT (1000 mg/kg) throughout 6 months of CS exposure resulted in a 63% improvement in airspace size and abolished CS-induced plasma tumor necrosis factor(TNF)-α increases [33]. In our study, a dose of 2 mg/kg of AAT-Fc and pAAT was administered, as this dosage of AAT-Fc showed effects in a previous study in mice [12]. A separate study showed that recombinant AAT administered by inhalation for 6 months prior to each day of CS exposure resulted in up to 73% reduction in airspace enlargement [34]. Smoking is a primary cause of emphysema in COPD and AATD patients, although only a subset of smokers develops COPD or emphysema [35, 36]. A variety of theories has been proposed to explain the underlying disease pathophysiology and include genetic susceptibility and exposure to poor ambient air quality at early ages [37]. Immune responses against components of lung tissue have been implicated [38]. Oligoclonal expansion of CD4^+^ T cells in lung tissue of emphysema patients has been shown [39] and immune responses against elastin as a component of lung tissue have been supported by increased production levels of IFNγ from peripheral blood CD4^+^ T cells in COPD patients [40]. Increases in IFNγ- and IL-6-producing blood T cells to elastin fragments had a positive association with the annual rate of emphysema progression in active smokers [41]. In addition, B-cells were up-regulated in patients with emphysema [42]. Such autoimmune responses towards lung tissue components are proposed to be a part of the pathogenesis of COPD and emphysema [20]. The findings that lung inflammation continues and lung damage progresses despite smoking cessation in severe cases of emphysema may support this notion of self-destructive processes [39]. The ongoing lung tissue damage after a single elastase administration also implicated autoimmune mechanisms, especially immunity against elastin fragments. Indeed, antagonizing elastin fragments inhibited the development of PPE-induced alveolar enlargement in mice [43].

A number of effects of pAAT were demonstrated in animal models beyond simple AAT replacement and restoration of anti-proteinase therapy with demonstrable anti-inflammatory and immunomodulatory activities. pAAT administration with a carrier lipoprotein prior to and following PPE instillation prevented increases in IL-6, monocyte chemoattractant protein 1, TNF-α, matrix metalloproteinase-2 and -9, fibronectin levels, and MLI [44]. An important target may be DCs as these cells are thought to play a role in the pathogenesis of COPD and emphysema [45]. DC numbers were increased in the small airways of COPD patients and the numbers were associated with disease severity [46–48]. Expression levels of the costimulatory molecule, CD83, on DCs in the lungs of COPD patients were also associated with disease severity [47, 48]. In animal models, DC activities were upregulated in PPE-induced [49] and CS-induced emphysema models, and following in vitro CS exposure [50–53]. pAAT treatment has been shown to modulate DC function with decreased MHC class II, CD40, and CD86 expression, reduced IL-6 release, and increased production of IL-10 [6]. pAAT treatment also inhibited LPS- and CpG-induced activation of DCs and reduced the production of inflammatory cytokines [54]. pAAT treatment altered DC function to become tolerogenic, whereas isolated T cell activities were not altered by pAAT treatment [55].

We determined the effects of AAT-Fc or pAAT on the antigen-presenting function of EP-primed bone marrow-derived DCs by determining their role in elastin-induced cytokine production following co-culture with spleen CD4^+^ (and CD8^+^) T cells derived from PPE-treated mice. CD4^+^ T cells from PPE-treated mice produced IFNγ and IL-17 following co-culture with EP-primed DCs. When CD4^+^ T cells were co-cultured with AAT-Fc treated DCs, cytokine production levels were decreased; cytokine production was less affected if the DCs were treated with pAAT.

Peptide-Fc fusion proteins are generated by fusing a biologically active peptide with the Fc-domain of immunoglobulin G to extend the half-life [13]. This is the result of the binding to the neonatal Fc receptor (FcγRn) as FcγRn is thought to protect their ligands [56]. In addition, the Fc portion of fused protein potently binds to Fcγ receptors which are expressed on many immune cells and modulate activities of these cells [13]. DCs also express Fcγ receptors and FcγRn [57]. Thus, AAT-Fc may be expected to promote desirable target cell binding and induce immunomodulatory activities more efficiently on immune cells, including DCs, compared to pAAT. An imbalance between protease and anti-protease activities is thought to be a primary mechanism in the pathogenesis of COPD including emphysema in AAT-sufficient patients (58). In light of the superior benefits of recombinant AAT-Fc over pAAT, the associated costs of pAAT vs. AAT-Fc, and the longer half-life of AAT-Fc, further development of AAT-Fc as a therapeutic tool in AAT-deficient and AAT-sufficient emphysema is warranted.

## Conclusions

We demonstrated the potent and superior therapeutic effects of AAT-Fc in elastase- and CS-induced models of emphysema when compared to pAAT. These effects were associated with immunomodulatory effects on DC activity. AAT-Fc may provide a therapeutic option to individuals with AATD- and CS-induced emphysema. Determining the clinical benefits of AAT-Fc in emphysema patients is warranted.

## Supplementary Information

**Additional file 1: Figure S1. ** AAT-Fc or control treatments were administered 1 day prior to hLE instillation and the effects in the lungs were analyzed 1 week later. Cst values (A), MLI (B), and S (C) were compared in mice that received saline instillation (saline), vehicle (PBS-PPE), AAT-Fc (AAT-Fc-PPE), or pAAT (pAAT-PPE) treatment prior to hLE. n = 8 in each group. *p < 0.05 by Tukey–Kramer test.

## Data Availability

All data generated or analyzed during this study are included in this published article and its supplementary information files. The datasets used and/or analyzed during the current study are available from the corresponding author on request.

## Abbreviations

AAT: Alpha-1 antitrypsin
AATD: Alpha-1 antitrypsin deficiency
AAT-Fc: Alpha-1 antitrypsin-IgG1 Fc-fusion protein
BAL: Bronchoalveolar lavage
CS: Cigarette smoke
Cst: Static compliance
EP: Elastin peptide
hLE: Human leucocyte elastase
i.p.: Intraperitoneal
MLI: Mean linear intercept
MNCs: Mononuclear cells
pAAT: Human plasma-derived alpha-1 antitrypsin
PPE: Porcine pancreatic elastase
Saline: Physiological saline solution
S: Alveolar surface area
TLC: Total lung capacity
