# Supplementary figures and images for: Therapeutic benefits of recombinant alpha1-antitrypsin IgG1 Fc-fusion protein in experimental emphysema
Source: Respir Res. 2021 Jul 16;22:207. doi: 10.1186/s12931-021-01784-y (PMC8283905; doi:10.1186/s12931-021-01784-y)

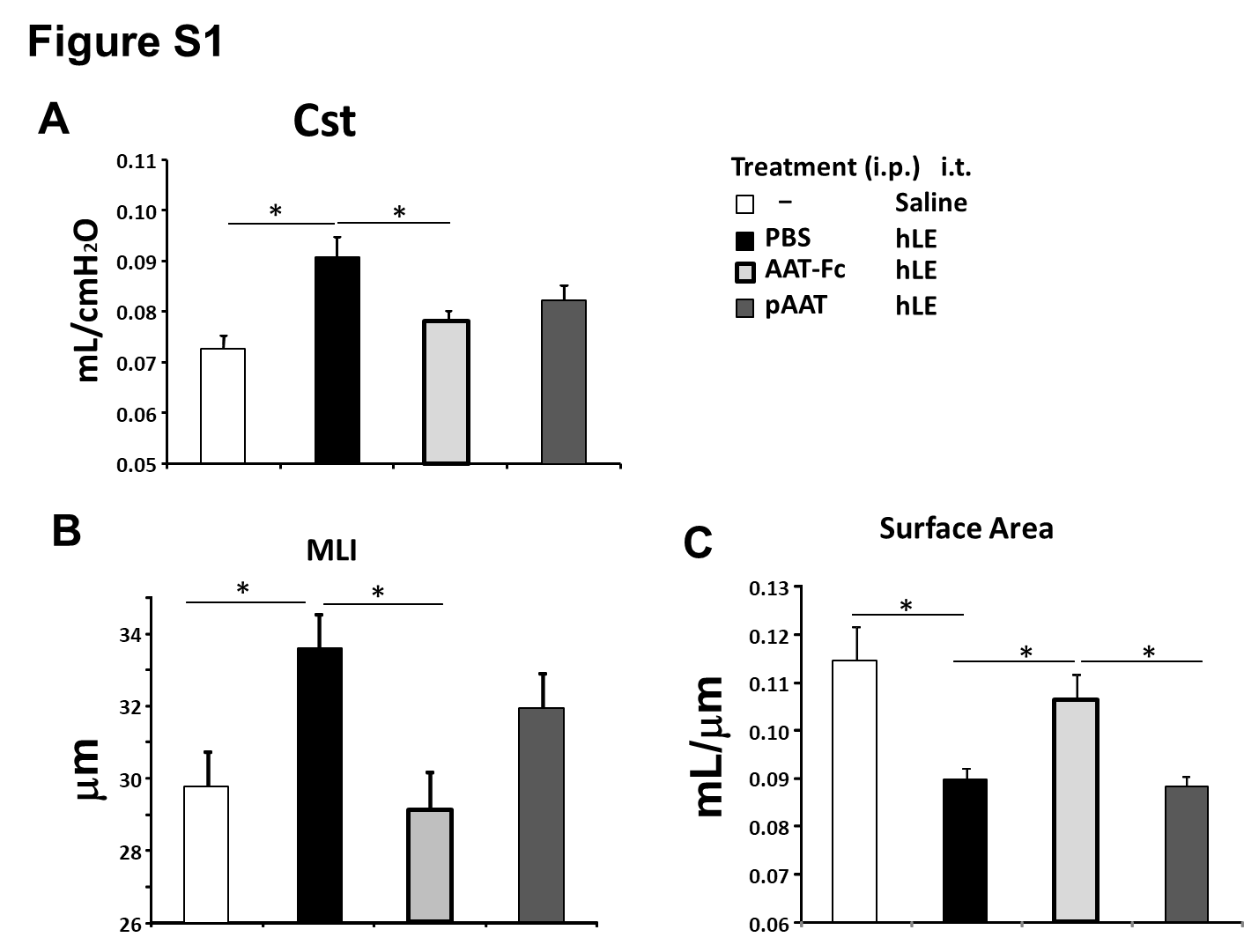

Supplement: Supplementary file 1 — Additional file 1: Figure S1. AAT-Fc or control treatments were administered 1 day prior to hLE instillation and the effects in the lungs were analyzed 1 week later. Cst values (A), MLI (B), and S (C) were compared in mice that received saline instillation (saline), vehicle (PBS-PPE), AAT-Fc (AAT-Fc-PPE), or pAAT (pAAT-PPE) treatment prior to hLE. n = 8 in each group. *p < 0.05 by Tukey–Kramer test. [file 12931_2021_1784_MOESM1_ESM.docx]
